# Integration of transcription and flux data reveals molecular paths associated with differences in oxygen-dependent phenotypes of *Saccharomyces cerevisiae*

**DOI:** 10.1186/1752-0509-8-16

**Published:** 2014-02-14

**Authors:** Erno Lindfors, Paula Jouhten, Merja Oja, Eija Rintala, Matej Orešič, Merja Penttilä

**Affiliations:** 1 VTT Technical Research Centre of Finland, Espoo, Finland; 2 Currently at: LifeGlimmer GmbH, Markelstrasse 38, D–12136 Berlin, Germany; 3 Currently at: Chemistry Building, Building 316, Dreijenplein 10, 6703 HB Wageningen, The Netherlands

**Keywords:** *Saccharomyces cerevisiae*, Network biology, Molecular path finding, Data integration, Oxygen, Constraint-based modeling

## Abstract

**Background:**

*Saccharomyces cerevisiae* is able to adapt to a wide range of external oxygen conditions. Previously, oxygen-dependent phenotypes have been studied individually at the transcriptional, metabolite, and flux level. However, the regulation of cell phenotype occurs across the different levels of cell function. Integrative analysis of data from multiple levels of cell function in the context of a network of several known biochemical interaction types could enable identification of active regulatory paths not limited to a single level of cell function.

**Results:**

The graph theoretical method called Enriched Molecular Path detection (EMPath) was extended to enable integrative utilization of transcription and flux data. The utility of the method was demonstrated by detecting paths associated with phenotype differences of *S. cerevisiae* under three different conditions of oxygen provision: 20.9%, 2.8% and 0.5%. The detection of molecular paths was performed in an integrated genome-scale metabolic and protein-protein interaction network.

**Conclusions:**

The molecular paths associated with the phenotype differences of *S. cerevisiae* under conditions of different oxygen provisions revealed paths of molecular interactions that could potentially mediate information transfer between processes that respond to the particular oxygen availabilities.

## Background

The transcriptome is a realization of the genome of an organism whereas the fluxes are an ultimate response of the complete multilevel regulatory system of a cell. The correlation between the transcriptome and the fluxes is usually weak [1] since a substantial part of the regulation of cell physiology occurs at the post-transcriptional and metabolic levels [2]. The regulation is mediated by interactions beyond individual levels of cell function. Active paths of regulatory interactions which determine the cell phenotype are concealed in data on cell components belonging to different regulatory levels. Integration of these data to frameworks of known interactions of multiple types could allow for a reconstruction of the regulatory paths associated with specific phenotypes. Genome-scale metabolic models build on the entity of metabolic enzyme encoding genes in the genome. These models are already available for various organisms and provide frameworks of metabolic interactions to the extent of whole cells. Metabolic network context is being utilized to identify transcriptionally differentially regulated pre-defined pathways of enzymes sharing metabolites as substrates and products by parametric gene set enrichment analysis [3]. Full interconnectivity of metabolism is being applied in the identification of reporter metabolites, regulatory hot spots around which the most significant transcriptional changes have occurred [4]. Protein-protein interactions facilitate various kinds of information transfer, e.g. a change in a localization or activity of a protein as a result of physical interaction or post-translational modification [5-7]. In particular, protein kinases serve as key regulators of nutrient sensing and signaling via protein-protein interactions. A network of interactions of key protein kinases of nutrient dependent regulation has been mapped, manually curated and annotated for the eukaryotic model organism *S. cerevisiae*[8]. A global network of protein kinase and phosphatase interactions that mediate information transfer via post-translational modifications is also available for *S. cerevisiae*[9] along with a large-scale data set on various types of physical protein-protein interactions [10].

Even other types of biochemical interactions, such as signaling and transcription factor interactions, also allow for communication between cellular components [11,12].

Previously, a graph-theoretical method called Enriched Molecular Path detection (EMPath) was developed in order to identify molecular interaction paths from multi-level interactome data [13]. The EMPath method was an extension of a “color coding” algorithm [14] which had earlier been used to detect signaling cascades based on edge reliabilities in protein-protein interaction networks [15] and more general structures, such as trees [16]. The developed EMPath method was applied to detect phenotype specific molecular paths in type 1 diabetes mouse models in an integrated network of metabolic, protein-protein and signal transduction interactions scored with transcription data [13]. Recently, several graph theoretical methods for detection of molecular paths in an interaction network context have been developed. Gene Graph Enrichment Analysis (GGEA) integrates a known gene regulatory network in an analysis of transcription data and gains interpretability of the regulation processes underlying the gene expression response [17]. FiDePa (Finding Deregulated Paths) [18] and Topology Enrichment Analysis frameworK (TEAK) [19] find differentially expressed pathways between two cell phenotypes in signaling or regulatory networks and metabolic pathways, respectively. A method called Clipper exploits network topology to detect signaling paths within longer pathways based on differential gene expression between two phenotypes [20]. However, all these methods employ a single type of phenotypic information (i.e. transcription data), whereas post-transcriptional regulation has a recognized and substantial effect on a phenotype. Therefore, the EMPath method was extended in this study to enable integrative simultaneous utilization of two data types, i.e. transcription and flux data in the context of a multi-level interaction network to detect enriched molecular paths associated with phenotypic differences.

Oxygen is a major determinant of physiology for the eukaryotic model organism *S. cerevisiae. S. cerevisiae* is able to remodel its energy generation and redox metabolism according to the availability of oxygen in such a flexible way that it can grow under a wide range of oxygen availabilities from fully aerobic conditions to anaerobiosis. Characterization of the oxygen-dependent phenotypes of *S. cerevisiae* has previously been reported at the individual transcriptional, metabolite, and flux levels [21-23]. In this study, two case-control settings of the oxygen dependent phenotype differences of *S. cerevisiae* were defined. The phenotype under conditions of 20.9% O_2_ provision was compared to the phenotype under conditions of 2.8% O_2_ provision, and the phenotype under conditions of 2.8% O_2_ provision was compared to the phenotype under conditions of 0.5% O_2_ provision. Previously, it was noted that *S. cerevisiae* had highly similar flux distributions under conditions of 20.9% and 2.8% O_2_ provision [23], but interestingly there were substantial differences in the transcriptomes [21]. The phenotypes of *S. cerevisiae* possessed substantially different flux distributions under conditions of 2.8% and 0.5% O_2_ provision [23], whereas the transcriptomes of the phenotypes were surprisingly similar [21]. Thus, transcription and flux data were integratively utilized to find enriched molecular interaction paths associated with the aforementioned differences in the previously observed oxygen-dependent phenotypes [21-23]. The path detection was performed in a combined network of metabolic [24-26] and protein-protein interactions (Search Tool for the Retrieval of Interacting Genes database (STRING): [27]) of *S. cerevisiae*.

## Methods

### Overview

**Figure 1:**
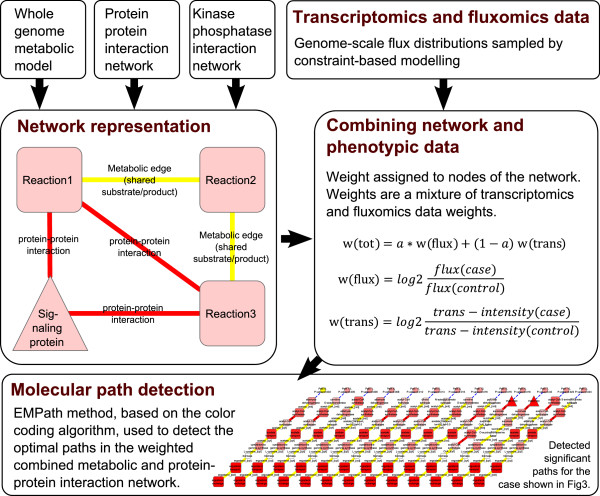
**Overall workflow of the study comprising the following main steps.** • genome-scale metabolic network model and protein-protein interactions, including kinase-phosphatase interactions, were integrated into single network representation. • phenotypic context from fluxome and transcriptome data incorporated into the network. • EMPath used for detecting up-and down-regulated paths. • detected paths were visualized and enriched with previously known functional categories.

Figure 1 illustrates the overall pipeline of the study. First, a genome-scale metabolic network model and the protein-protein interactions including the global kinase-phosphatase interactions [9] were integrated into a single interaction network. Then, flux and transcription data were assigned to node weights to set the network into a phenotypic context. Then, the EMPath method was used to detect enriched up- and down-regulated molecular interaction paths within the network. In the end, the paths were visualized as integrated networks and enriched with previously known functional categories.

### Network representation

The integrated network of metabolic and protein-protein interactions comprised of a recently refined version [24] of the yeast whole genome metabolic model, protein-protein interactions from the STRING database [27], and a kinase-phosphatase interaction network [9]. From the STRING database the protein interactions with an experimental score greater than 900 were included, thus excluding interactions with low experimental evidence. The integrated network representation is illustrated in Figure 1. In this representation the metabolic reactions of the genome-scale model [24] are nodes and there is an edge between two reactions if they share a metabolite, i.e. having either a common substrate or product. Cofactors and other metabolites not participating in the metabolic conversions with their carbon backbone were excluded from the network. The excluded metabolites are listed in Additional file 1. All edges were modeled with undirected edges. Each reaction comprised a set of gene(s) that encodes an enzyme that catalyzes the reaction. Protein-protein interactions were integrated with nodes representing enzymatic reactions if the metabolic enzymes had reported protein-protein interactions. In total, the whole integrated network comprised 5 702 nodes and 41 525 edges.

### Transcription and flux data

Wiebe et al. (2008) grew *S. cerevisiae* in glucose-limited chemostat cultivations at a dilution rate of 0.1 h^-1^ under different oxygenation conditions (i.e. 20.9%, 2.8%, 1.0% and 0.5% O_2_) in the chemostat inlet gas to obtain the oxygen dependent phenotypes [22]. Rintala et al. (2009) performed the analysis of the transcriptomes of *S. cerevisiae* under the different conditions of oxygen provision [21]. The normalized transcription dataset was stored in the Gene Expression Omnibus (GEO) database [28] with the accession number GSE12442. In the present study, all four replicates of transcription data from each of the steady state cultures with 20.9%, 2.8%, and 0.5% O_2_ in the chemostat inlet gas were used to determine the transcription scores for the detection of molecular paths.

Genome-scale flux distributions were sampled from the solution space of a genome-scale metabolic model of *S. cerevisiae* by Monte Carlo sampling using Artificial Hit-And-Run (ACHR) sampler [29]. Prior to the sampling, the genome-scale metabolic model of *S. cerevisiae* was improved by further refinement of its oxygen dependent metabolism [24] (Additional file 1). The model was also constrained with P/O ratios dependent on a specific oxygen uptake rate (OUR) [23] and experimental data reported on extracellular fluxes, i.e., growth rate, substrate consumption rates and product secretion rates [22]. The Carbon Evolution Rate (CER), resulting from carbon dioxide production at various sites in metabolism, was allowed to vary freely to introduce flexibility to the system since the remaining secretion rates were set to zero. However, the introduction of the exact experimental rate constraints resulted in an infeasible solution space. Thus, the lower and upper bound constraints derived from the extracellular growth, glucose uptake, and ethanol secretion rates were simultaneously and gradually expanded until a feasible flux solution existed. At each step the constraints were expanded with 10% of the particular SEMs (Standard Error of the Mean) of the extracellular rates [22] (see Additional file 1 for the final constraints). OUR and P/O ratio constraints were kept as strict constraints since the oxygen uptake rates followed from the provision of oxygen in the chemostat inlet gas, which was the only experimental parameter changed in the bioreactor cultivations resulting in the three different phenotypes of *S. cerevisiae*[22] that were investigated in this study. Further, P/O ratios of *S. cerevisiae* dependent on OUR were previously determined [23] and used here. The Monte Carlo sampling of flux distributions was performed with the ACHR sampler [29] implemented in the COBRA Toolbox [30]. A threshold for the reactions with non-zero fluxes was set to a minimum of 10^-7^ mmol/(g CDW h). Zero fluxes were assigned to the rest of the reactions. A total of 10 000 feasible points were collected in the solution space out of which 2 000 samples were randomly selected for the calculation of mean fluxes. The mean values of unconstrained CER in the flux distribution samples differed from 4% to 13% from the experimental values.

### Combining network and phenotypic data

Previously, only transcription data was used as phenotypic data in the detection of enriched molecular paths [13]. Here the EMPath method was extended for integrative utilization of transcription and flux data having separate weights: *w*(*trans*), and *w*(*flux*), respectively. More specifically *w*(*trans*) is defined in Formula (1) in which *trans* - *intensity*(*case*) and *trans* - *intensity*(*control*) are case and control intensities of mRNA expression level averaged over all replicates, respectively. In the genome-scale metabolic model of *S. cerevisiae* the gene regulatory rules are expressed by AND-and OR-operands for the metabolic reactions (e.g. multi-protein complex as catalyst) that have more than one encoding gene [25]. If there was an OR-operand between two genes, then a mean intensity was calculated and if there was an AND-operand, then a minimum intensity was taken. Since there is no transcriptome data for non-enzymatic reactions (i.e. they do not require a catalyzing enzyme or an encoding gene to occur), neutral weights (i.e. zero) were assigned for them.

(1) $$w\left(\mathrm{trans}\right)=\log2\frac{\mathrm{trans}-\mathrm{intensity}\left(\mathrm{case}\right)}{\mathrm{trans}-\mathrm{intensity}\left(\mathrm{control}\right)}$$

The weight derived from the flux data for each reaction, *w*(*flux*), is defined in Formula (2) in which *flux*(*case*) and *flux* (control) were obtained by calculating averages over the 2 000 randomly selected samples, each corresponding to a feasible flux distribution (see Transcription and Flux data above).

(2) $$w\left(\mathrm{flux}\right)=\log2\frac{\mathrm{flux}\left(\mathrm{case}\right)}{\mathrm{flux}\left(\mathrm{control}\right)}$$

The total score for the node is defined in Formula (3). When the two data types were simultaneously used, *w*(*trans*) and *w*(*flux*) were scaled to be in the same interval, which was essential to prevent either of them from being over-represented in the detected molecular paths. In practice, the flux data was scaled to have the same range as the transcription data: {-2.71, 4.75} for 2.8% vs. 0.5% oxygen in the bioreactor inlet gas and {-3.31, 4.97} for 20.9% vs. 2.8% in the bioreactor inlet gas. Flux data was naturally not available for signaling proteins (i.e. non-metabolic proteins), thus their scores were calculated solely from the transcription data.

(3) $$\begin{array}{cc} w\left(\mathrm{tot}\right) & =a*w\left(\mathrm{flux}\right)+\left(1-a\right)*w\left(\mathrm{trans}\right),\\ \;a & =\left\{0,,,0.5,,,1\right\} \end{array}$$

The motivation of using parameter *a* was to allow for relative weighting for the flux and transcription data in the detection of molecular paths e.g. weighting with pure transcription data: a = 0, or pure flux data: a = 1, or their simultaneous utilization with an equal weight: a = 0.5.

### Molecular path detection

After the weights were assigned to the nodes, the EMPath method [13] was used to detect an optimal path of length *k*. The algorithm is initialized by assigning colors, i.e. random integer numbers [1, *k*], to the nodes of the path. Then a node with a maximum weight score is added to be the first node in the path. Then the neighboring nodes to the recently added node are considered to be the next node in the path. From this set a node with a maximum weight score is added to the path but nodes with a color that is already included in the path are ignored. Nodes are added until there are *k* nodes in the path. Then a score of the path is calculated by summing up all the node weights.

In order to calculate the p-value for the null hypothesis (i.e. that the detected path is obtained by chance), a random distribution was created by shuffling the node weights 1 000 times. After each shuffle, a path was detected and its score was calculated as described above. In this way, 1 000 optimal path scores in a random network were obtained resulting in a random distribution. A p-value for the null hypothesis that the detected path is obtained by chance was defined by comparing its score to the random distribution. 0.025 was used as a cut-off p-value, i.e. paths of higher p-values were not considered significant. A network was considered *harvested* from optimal paths if there were *i* consecutive iterations in which the detected path was detected during previous iterations.

The path detection was performed separately for up-and down-regulated paths in both case-control comparisons (20.9% vs. 2.8%, and 2.8% vs. 0.5% O_2_ in the bioreactor inlet gas), and for each value of parameter *a* ∈ {0, 0.5, 1}. When the up-regulated paths were detected, case-control ratios were used, and when the down-regulated paths were detected, control-case ratios were used. Eight (8) was used as the path length *k*. There is not any rigorous way to define the proper value for this parameter. Eight (8) was empirically found to be a proper value for this parameter: smaller values (e.g. 7) led to too sparse combined networks of enriched molecular paths and higher values (e.g. 9) led to very dense combined networks of enriched molecular paths which would have had poor interpretability. In similar vein, ten (10) was selected for parameter *i* on empirical basis: the higher values did not harvest the network significantly more thoroughly. The path detection calculations were implemented in a C++ environment and were processed on an Ubuntu Linux Server with 2 processors of Intel Xeon X5650 2.66 GHz divided in 24 virtual cores and 70 GB of RAM memory.

### Enrichment of functional protein categories

In order to study how pre-established cellular functions were associated with the detected molecular paths, the combined networks were associated with functional protein categories from FunCat [31] by making a hypergeometric test with controlling false discovery rate (FDR) [32] q-value 0.05 as a cut-off, as described in [13]. Open reading frame identifiers (ORF) were used to identify the genes.

### Path length

The method required a selection of pre-defined path length, which is heuristic and deserves some discussion. Let us assume that the network comprises *n* nodes, and for simplicity they are assumed to be fully connected to each other. In this case the network comprises $\left(\begin{array}{c} n\\ k \end{array}\right)$ paths of length  *k*, in which  *n* ≫ *k*. The higher the length *k* is the more paths the network comprises. Thus, a too small path length would lead to information poor networks. On the other hand, a drawback of a long path length is that the computational enumeration and the interpretation of crowded combined networks gets heavy. Eight was selected as the path length since it is the shortest length that provides paths which reasonably combine both metabolic and protein-protein interactions in all the studied cases.

## Results and discussion

### Effect of relative weighting of transcription and flux data on the detected molecular paths

The detected molecular interaction paths combined protein-protein interactions and metabolic interactions dependent on the phenotypes compared and the relative weighting used to combine the transcription and flux data. The numbers of protein-protein interactions (PPI) and metabolic edges in the combined networks of the detected molecular paths for each of the phenotype comparisons are shown in Table 1. Metabolic edges prevailed when a = 1 (i.e. only flux data used) in all comparisons except “2.8% vs. 0.5%, down” where there were as many PPI edges as metabolic edges. When the metabolic edges prevailed the detected paths generally followed the metabolic routes in which the fluxes had changed substantially. The neighboring metabolic reactions had correlated flux weights as the result of the steady state flux data being constrained by metabolic network stoichiometry. There were two comparisons (“2.8% vs. 0.5%, down” and “20.9% vs. 2.8%, down”) in which PPI edges prevailed when a = 0 (i.e. only transcription data used) indicating that in these comparisons metabolic pathways were less coherently transcriptionally down-regulated than the paths following protein-protein interactions.

**Table 1:** Size information of detected paths and combined network in each comparison

**Comparison (percentage of oxygen in the bioreactor inlet gas)**	**# PPI edges**	**# Metabolic edges**	**% PPI edges**	**% metabolic edges**	**# nodes**	**# paths**
2.8% vs. 0.5% O_2_, down, a = 0 (only transcription data)	20	3	87	13	12	12
2.8% vs. 0.5% O_2_, down, a = 0.5 (both transcription and flux data)	14	17	45	55	23	27
2.8% vs. 0.5% O_2_, down, a = 1 (only flux data)	14	14	50	50	14	23
2.8% vs. 0.5% O_2_, up, a = 0 (only transcription data)	4	39	9	91	29	23
2.8% vs. 0.5% O_2_, up, a = 0.5 (both transcription and flux data)	4	32	11	89	30	31
2.8% vs. 0.5% O_2_, up, a = 1 (only flux data)	1	25	4	96	16	15
20.9% vs. 2.8% O_2_, down, a = 0 (only transcription data)	20	17	54	46	27	18
20.9% vs. 2.8% O_2_, down, a = 0.5 (both transcription and flux data)	3	17	15	85	12	13
20.9% vs. 2.8% O_2_, down, a = 1 (only flux data)	1	17	6	94	17	18
20.9% vs. 2.8% O_2_, up, a = 0 (only transcription data)	15	51	23	77	42	42
20.9% vs. 2.8% O_2_, up, a = 0.5 (both transcription and flux data)	2	23	8	92	21	13
20.9% vs. 2.8% O_2_, up, a = 1 (only flux data)	35	42	45	55	43	64

Column 1: The number of PPI edges.

Column 2: The number of metabolic edges.

Column 3: The fraction of PPI edges from all edges.

Column 4: The fraction of metabolic edges from all edges.

Column 5: The number of nodes in the combined network.

Column 6: The number of detected paths.

### Peroxisomal activities and oxidative stress response featured in the upregulated interaction paths of phenotype differences between the fully respirative phenotype of *S. cerevisiae* and the respirofermentative phenotype at 2.8% oxygenation

**Figure 2:**
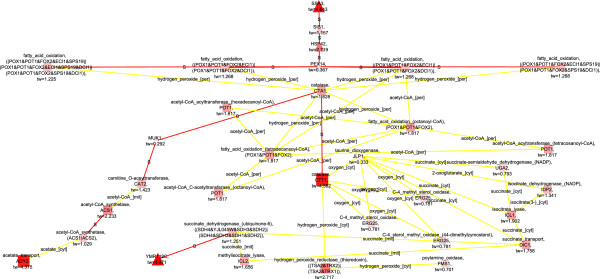
**Detected up-regulated molecular paths combined into one network, 20.9% vs 2.8%**_**, **_only transcription data used^*^.

Wiebe et al. (2008) had previously observed that the metabolism of *S. cerevisiae* was fully respirative under conditions of 20.9% O_2_ in the bioreactor inlet gas whereas under conditions of 2.8% O_2_ in the bioreactor inlet gas the metabolic state was respirofermentative [22]. However, the drop in the specific Oxygen Uptake Rate (OUR) was small, from 2.7 ± 0.04 to 2.5 ± 0.04 mmol/(g CDW h) [22] and Jouhten et al (2008) observed that the flux distributions remained almost constant except for the subtle flux to ethanol production [23]. Nevertheless, major changes between the two phenotypes have been observed at the transcriptional level [21]. The transcription and flux data for *S. cerevisiae* during steady state growth conditions at 20.9% and 2.8% oxygen provision were analyzed here in an integrative manner and separately with the EMPath method to detect molecular interaction paths that were possible determinants of the phenotypic differences observed in *S. cerevisiae* growing under the two different oxygenation conditions. When transcription data on *S. cerevisiae* growing under fully aerobic conditions and under conditions of 2.8% O_2_ in the bioreactor inlet gas was solely used in the scoring of the up-regulated nodes in the detection of molecular interaction paths, cellular processes of respirative metabolism, fatty acid oxidation, and oxidative stress defense were represented in the paths (Figure 2, FunCat enrichments in Additional file 1). Glyoxylate pathway enzyme isocitrate lyase encoded by *ICL1* and a dicarboxylate carrier transporting succinate from glyoxylate cycle into mitochondria to be incorporated into TCA cycle encoded by *DIC1*[33] appeared in the molecular paths up-regulated at the level of gene expression. The glyoxylate cycle is known to be induced in *S. cerevisiae* under respirative conditions for the metabolism of non-fermentative carbon sources [34]. In addition, the methylisocitrate lyase reaction catalyzed by an enzyme encoded by *ICL2*, which is homologous to *ICL1*, was also included in the detected molecular paths. Isocitrate dehydrogenase encoding *IDP2* was connected via isocitrate to isocitrate lyase of the glyoxylate cycle. The *IDP2* encoded isoform is an alternative source of cytosolic NADPH, for the pentose phosphate pathway, but only while the metabolic state is respirative [35]. Succinate interconnected the glyoxylate cycle components further to *SHH3* (YMR118C) (fold change 5.0) encoding a putative mitochondrial inner membrane protein [36]. *SHH3* was linked via a protein-protein interaction to ubiquinone-6 dependent succinate dehydrogenase. Succinate dehydrogenase was expectedly the only respiratory chain coupled component observed since most of the respiratory chain components in *S. cerevisiae* are expressed on a lower level under fully aerobic conditions than in conditions of lower oxygen provision [21]. In addition to the respirative metabolism, fatty acid beta oxidation was observed in the detected molecular paths. Beta oxidation of fatty acids occurs in peroxisomes in yeast and provides an alternative energy source for *S. cerevisiae* under aerobic conditions. Accordingly, *PEX14*, which is involved in the import of peroxisomal proteins [37], had protein-protein interactions with the components of fatty acid beta oxidation in the detected paths. Both peroxisome biogenesis and fatty acid beta oxidation are under regulation by SNF1p kinase, a coordinator of energy metabolism of *S. cerevisiae*[38]. The transcriptional regulation of the peroxisome biogenesis and fatty acid beta oxidation also involves the common regulators ADR1p, OAF1p, and PIP2p. Rintala *et al.* (2009) showed that the genes involved in fatty acid beta oxidation and peroxisomal biogenesis were expressed at higher levels under the fully aerobic conditions than in conditions of any lower oxygen provision [21]. In the detected molecular interaction paths *PEX*14 was further linked to regulators of protein folding (*HSP42*, *SIS1*, *SSA3*) in particular in response to stress, which share a YAP1p binding site [YEASTRACT database July 16, 2013; [39-41]]. YAP1p is a transcription factor responsive to oxidative stress. In the detected molecular paths fatty acid beta oxidation was connected to oxidative stress defense via *CTA1* which encodes for a catalase required for the removal of hydrogen peroxide, a strong oxidant, in the peroxisomal matrix. Hydrogen peroxide is formed as a byproduct in the beta oxidation of fatty acids. CTA1p was further linked to a cytosolic catalase reaction involved in the defense against oxidative damage encoded by *CTT1* (fold change 4.6) and a hydrogen peroxide reductase reaction that mediates the maintenance of cellular redox balance. Koerkamp et al. (2002) has observed an induction of peroxisomal fatty acid oxidation to trigger transient YAP1p mediated oxidative stress response [42]. However, the transient oxidative stress response did not induce an expression of *CTT1* and *CTA1* co-responded non-transiently with other genes involved in the peroxisomal functions. Here, the up-regulation of the defense against oxidative agents linked to the up-regulation of peroxisomal activities via molecular interaction paths in *S. cerevisiae* cells provided with air compared to cells provided with 2.8% oxygen in the chemostat inlet gas, suggests that *S. cerevisiae* co-regulates these activities. The peroxisomal activities and oxidative stress defense could be down-regulated either directly in response to the decreased oxygen availability though it did not result in substantially lowered oxygen uptake rate (2.7 mmol/(g CDW h) vs 2.5 mmol/(g CDW h) under provision of 20.9% vs 2.8% oxygen, respectively [22]), or in response to the induced fermentative metabolism in cells provided with 2.8% oxygen in the chemostat inlet gas.

Acetyl-CoA synthesis and shuttling were interconnected to the *CTT1* encoded catalase and defense against oxidative agents via protein-protein interactions and a guanine nucleotide exchange factor MUK1p which is involved in protein trafficking [43]. MUK1p had a protein-protein interaction to carnitine o-acetyltransferase of the carnitine shuttle which is active both in peroxisomes and in mitochondria. The carnitine shuttle transfers acetyl-CoA across peroxisomal and mitochondrial membranes. *CAT2* encodes the carnitine o-acetyltransferase in *S. cerevisiae* and was coupled to an acetyl-CoA synthetase isoform encoded by *ACS1*, which is induced under respirative metabolism in *S. cerevisiae*[44]. *ACS1* was down-regulated when 2.8% O_2_ was provided compared to fully aerobic conditions, even though the metabolism of *S. cerevisiae* was mainly respirative. The localization of the *ACS1* encoded acetyl-CoA synthetase has been very unclear until recently when Chen *et al.* (2012) confirmed at least a distributed localization of the *ACS*1 encoded enzyme between cytosol and peroxisomes [45]. However, ACS1p has also been observed in the mitochondrial proteome [46]. Perhaps the down-regulation of *ACS1* in response to the subtle decrease in the oxygen uptake rate under conditions of 2.8% O_2_ provision was related to a general down-regulation of the peroxisomal activities. Remarkably, the decreased oxygen provision which resulted in a mild decrease in the respiratory activity [21-23] triggered the down-regulation of peroxisomal functions coupled to the fatty acid beta oxidation whereas a respiratory deficiency in an absence of oxygen limitation has been observed to trigger an opposite response, an up-regulation of peroxisomal activities [47].

**Figure 3:**
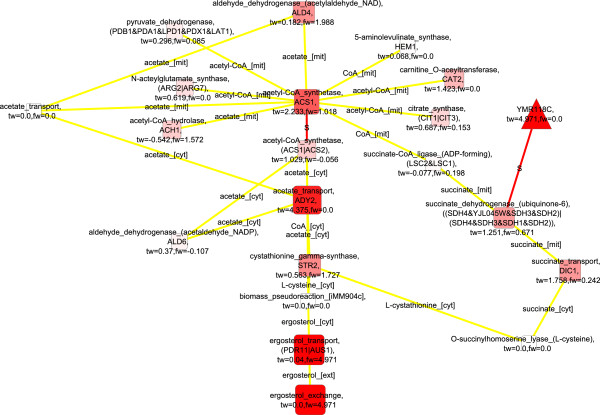
Detected up-regulated molecular paths combined into one network, 20.9% vs 2.8, both transcription and flux data used^*^.

When both transcription and flux data were used to score the nodes of the network in the EMPath method, the molecular paths up-regulated in the fully respirative phenotype of *S. cerevisiae* compared to the respirofermentative phenotype observed under 2.8% oxygenation [22] included key enzymes of respirative metabolism i.e. pyruvate dehydrogenase, the gate keeper of the TCA cycle, and citrate synthase (Figure 3, FunCat enrichments in Additional file 1). They were linked to the *ACS1* encoded acetyl-CoA synthetase which was observed in the enriched molecular paths when the path detection was run solely with the transcription data. Further connections were observed to the mitochondrial NAD^+^ dependent and cytosolic NADP^+^ dependent isoforms of acetaldehyde dehydrogenase encoded by *ALD4* and *ALD6*, respectively [48,49]. Both the *ALD4* encoded isoform and the *ALD6* encoded isoform, which is an additional source of cytosolic NADPH, had lower mRNA and protein levels under oxygen limitation than under fully aerobic conditions [21]. The mRNA and protein levels of *ALD4* and *ALD6* encoded acetaldehyde dehydrogenase isoenzymes correlated within five different conditions of oxygen provision from fully aerobic to anaerobic. Here flux estimation also suggested changes in the fluxes of the reactions catalysed by both isoforms. The succinate dehydrogenase reaction, which is closely coupled to the respiratory chain, showed an altered flux response between the compared conditions and was observed in the detected paths when only the transcription data was used in scoring. However, the glyoxylate cycle components and components involved in the peroxisomal fatty acid beta oxidation were absent from the molecular paths when the flux data was included in the scoring. The glyoxylate cycle is under glucose repression [34] and no *in vivo* activity of the glyoxylate cycle in *S. cerevisiae* was previously observed in the ^13^C-labelling experiments on glucose either under fully aerobic conditions or in 2.8% oxygenation [23].

Scoring the nodes of the interaction network solely with flux data resulted in molecular interaction paths dominated by components of sphingolipid metabolism and protein-protein interactions between them (Additional file 2: Figure S1; FunCat enrichments in Additional file 1). Expression of *SUR2* and *SCS7* encoded hydroxylases involved in the biosynthesis of sphingolipids has been found to be oxygen-dependent [50,51]. Thus, OUR may have had an effect on the *in vivo* activity of the sphingolipid biosynthesis pathway. Sphingolipid metabolism has been associated with ageing and apoptosis [52] which were observed in the FunCat enrichments of the detected molecular paths.

### Downregulated interaction paths of phenotype differences between fully respirative phenotype of *S. cerevisiae* and respirofermentative phenotype at 2.8% oxygenation involved regulation of the cell cycle at the transcriptional level

**Figure 4:**
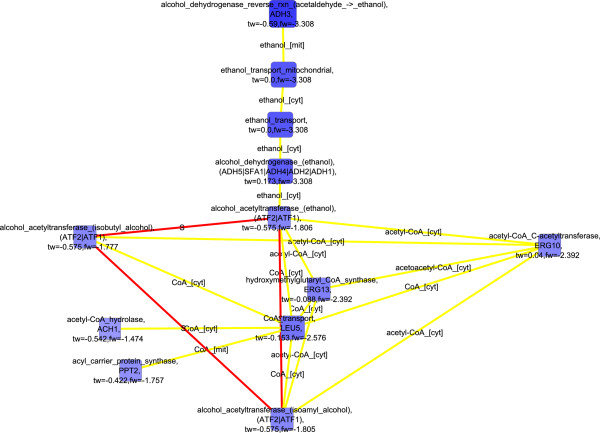
Detected down-regulated molecular paths combined into one network, 20.9% vs 2.8%, both transcription and flux data used^*^.

**Figure 5:**
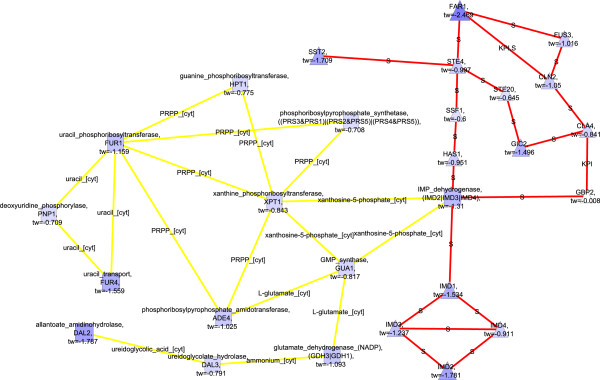
Detected down-regulated molecular paths combined into one network, 20.9% vs 2.8%, only transcription data used^*^.

Components of fermentative metabolism, alcohol dehydrogenases in particular, were present in the down-regulated molecular paths in the fully respirative phenotype of *S. cerevisiae* compared to the respirofermentative phenotype of *S. cerevisiae* under the 2.8% oxygenation conditions when both transcription and flux data were incorporated into the scores (Figure 4, both transcription and flux data used in the scoring; Additional file 2: Figure S2, scoring with pure flux data; FunCat enrichments in Additional file 1). When only transcription data was used in the scoring, a separate, interconnected, network of regulatory components was observed (Figure 5). The regulatory components were involved in the mating pathway and in the regulation of the cell cycle (FunCat enrichments in Additional file 1). The separate regulatory network was linked via protein-protein interactions to IMP dehydrogenase and, thus, to nucleotide synthesis.

Notably, alcohol dehydrogenase was found in the detected molecular paths only when flux data was included in the scoring even though alcohol production was a major phenotypic difference between *S. cerevisiae* under fully aerobic and conditions or 2.8% oxygen provision. This emphasizes the benefit of integrated data from a post-transcriptional regulatory level into the analysis.

### Upregulated molecular interaction paths detected in *S. cerevisiae* between the respirofermentative phenotypes at 2.8% oxygenation and 0.5% oxygenation suggest remodelling of transport across the mitochondrial membrane

**Figure 6:**
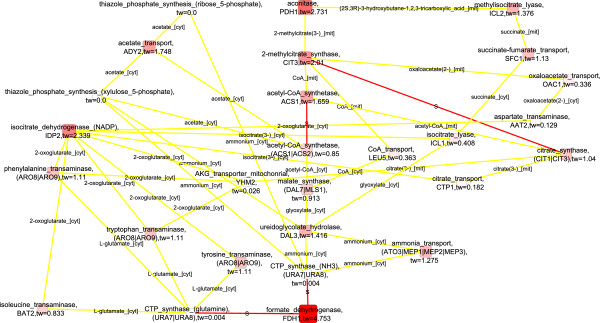
Detected up-regulated molecular paths combined into one network, 2.8% vs 0.5%, only transcription data used^*^.

The metabolic state of *S. cerevisiae* was respirofermentative under both conditions: 2.8% and 0.5% O_2_ in the bioreactor inlet gas [22] and the transcriptomes of *S. cerevisiae* were observed to be similar under these two conditions [21]. However, the flux distributions were substantially different [23]. Under the 0.5% oxygenation conditions the yield of ethanol on glucose exceeded the yield of biomass on glucose, and pyruvate decarboxylase carried the main flux from pyruvate branching point in contrast to the subtle ethanol production of *S. cerevisiae* under 2.8% oxygenation conditions [23]. The detected molecular paths up-regulated in *S. cerevisiae* under the 2.8% oxygenation conditions compared to the 0.5% oxygenation conditions when the transcription data was solely used to score nodes, featured a remodeling of transport between the cytosol and mitochondria, and respirative metabolism (Figure 6; FunCat enrichments in Additional file 1). The remodelling of respirative metabolism at the transcriptional level was progressive as a function of oxygenation since the glyoxylate cycle components and *ACS1* encoded acetyl-CoA synthetase and isocitrate dehydrogenase encoded by *IDP2* were observed also in the molecular paths representing the differences of the response of *S. cerevisiae* to fully aerobic conditions and conditions of 2.8% oxygen provision. The glyoxylate cycle was represented in the molecular paths detected for the differences of *S. cerevisiae* phenotypes within 2.8% and 0.5% oxygenation conditions by both malate synthase encoded by *MLS1* and isocitrate lyase. In addition, components of the propionate catabolic pathway, which resembles the glyoxylate cycle, including a 2-methylcitrate synthase encoded by *CIT3*, aconitase encoded by *PDH1*, and methylisocitrate lyase encoded by *ICL2* were observed in the paths. Methylisocitrate lyase cleaves methylisocitrate into succinate and pyruvate which integrate to the TCA cycle. Propionate catabolism is generally under glucose repression [53] but *PDH1* has also been observed to be regulated by retrograde regulators and induced in mitochondrial dysfunction [47]. However, here, during decreased respiratory activity due to a limited availability of oxygen, *PDH1* was down-regulated. Interestingly, a number of transports between the cytosolic and mitochondrial compartments were observed in the detected molecular paths. The transporters were carriers of the intermediates of TCA cycle, and acetate and CoA. Proton gradient across the mitochondrial membrane affects the molecule and ion transport since many of the transporters are proton symporters or antiporters. The appearance of the transporters in the up-regulated molecular paths suggests that in 0.5% oxygenation conditions the low availability of oxygen may have limited the generation of proton gradient across the mitochondrial membrane by the electron transfer chain of *S. cerevisiae* and, thus, the transport required reorganization.

**Figure 7:**
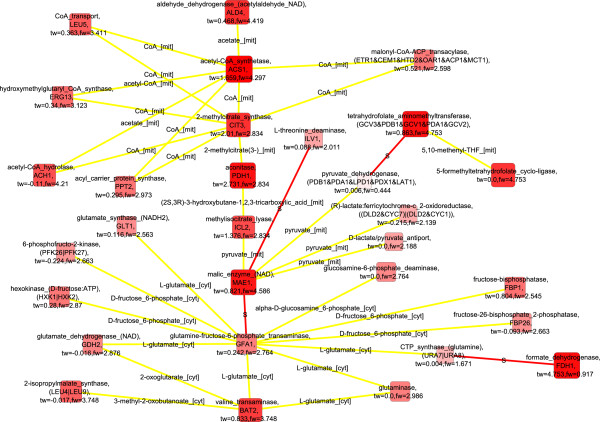
Detected up-regulated molecular paths combined into one network, 2.8% vs 0.5%, both transcription and flux data used^*^.

When both transcription and flux data were used in the scoring of nodes up-regulated in *S. cerevisiae* under the 2.8% oxygenation conditions compared to the 0.5% oxygenation, additional components involved in aerobic metabolism such as fructose 6-phosphatase, a gluconeogenetic enzyme, encoded by *FBP1* and pyruvate dehydrogenase complex were observed among others (Figure 7; FunCat enrichments in Additional file 1). Again, the glyoxylate cycle components were absent when flux data was included in the scoring whereas the components involved in propionate metabolism were observed.

Mevalonate biosynthesis prevailed in the detected up-regulated molecular paths when only flux data was used to score the nodes (Additional file 2: Figure S3; FunCat enrichments in Additional file 1). In addition, acetaldehyde dehydrogenase isoforms encoded by *ALD4* and *ALD5* catalyzing the mitochondrial NADP^+^ specific and cytosolic NAD^+^ specific reactions were observed. Most of the metabolic interactions in the detected paths involved either acetyl-CoA or CoA.

### Potential post-transcriptionally co-regulated reactions found in the downregulated molecular interaction paths detected in *S. cerevisiae* between the respirofermentative phenotypes at 2.8% oxygenation and 0.5% oxygenation

**Figure 8:**
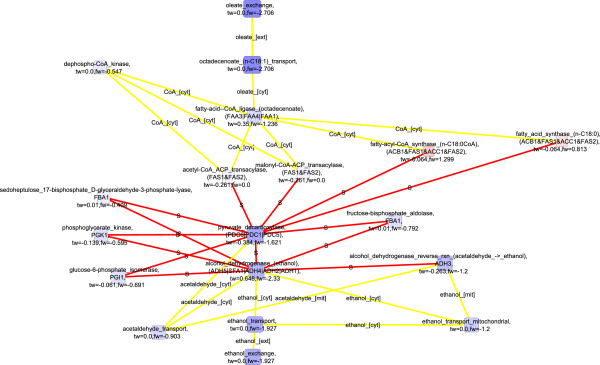
Detected down-regulated molecular paths combined into one network, 2.8% vs 0.5%, both transcription and flux data used^*^.

**Figure 9:**
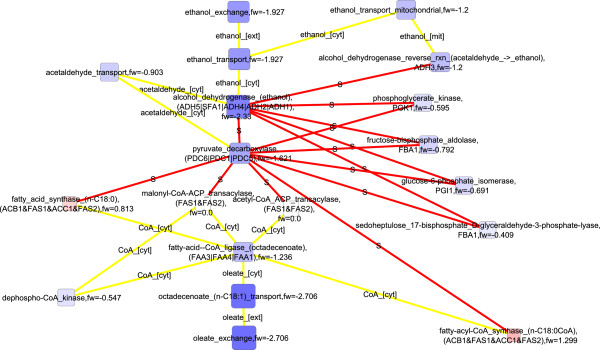
Detected down-regulated molecular paths combined into one network, 2.8% vs 0.5%, only flux data used^*^.

When both flux and transcription data were used in the scoring of nodes down-regulated in *S. cerevisiae* under the 2.8% oxygenation compared to the 0.5% oxygenation, key enzymes of the central carbon metabolism, glucose-6-phosphate isomerase, fructose bisphosphate aldolase, phosphoglycerate kinase, pyruvate decarboxylase, and alcohol dehydrogenase were observed in the detected molecular paths (Figure 8). These enzymes, involved in the glycolytic pathway, pyruvate metabolism, and fermentative pathway (FunCat enrichments in Additional file 1), are not directly linked by metabolic interactions, but were connected by protein-protein interactions in the detected molecular paths. Collins *et al.* (2007) reported in their high-throughput study the protein-protein interactions between glucose 6-phosphate isomerase (PGI1p), fructose bisphosphate aldolase (FBA1p), 3-phosphoglycerate kinase (PGK1p), pyruvate decarboxylase (PDC1p), and alcohol dehydrogenase (ADH1p) [54]. The genes encoding the discussed enzymes, i.e. *FBA1, PGK1, PDC1,* and *ADH1*, have all been observed to have stable expression under a range of conditions [55]. However, the fluxes of glucose 6-phosphate isomerase, fructose bisphosphate aldolase, 3-phosphoglycerate kinase, pyruvate decarboxylase, and alcohol dehydrogenase reactions were substantially lower under 2.8% oxygenation conditions than under even lower oxygen availability [23] whereas the corresponding transcript levels did not, as expected, show consistent behavior [21]. On the other hand, the level of FBA1p is under post-transcriptional control by 14-3-3 proteins BMH1p and BMH2p [56]. In fact, post-transcriptional regulation was previously observed to have a major effect on the protein levels in *S. cerevisiae* under the conditions of 0.5% O_2_ in the bioreactor inlet gas [21]. If the physical interactions between these enzymes mediate a transfer of information in some form, they enable coordinated regulation of the central carbon metabolism in upper and lower glycolysis, and in the fermentative pathway. The information transfer could occur for example via a common post-translational modification occurring while the proteins interact. Notably, all these enzymes contain identified phosphorylation sites (http://www.phosphopep.org) [57] and a differential phosphorylation of one of the enzymes, fructose bisphosphate aldolase (FBA1p), in response to switch in growth conditions was recently observed by Oliveira *et al.* (2012) [58]. Protein-protein interactions interconnected the enzymes of central carbon metabolism further to fatty acid import and biosynthesis. The detected molecular interaction paths included *FAS1* and *FAS2* that are involved in the elongation of saturated fatty acids, and *FAA1* and *FAA4* encoding enzymes catalyzing the import and activation of unsaturated fatty acids available in the growth medium. The detected down-regulated molecular paths were highly similar involving the components of the central carbon metabolism when pure flux data was used in the scoring (Figure 9). If flux data was not incorporated into the scoring, only amino acid transport was observed (Additional file 2: Figure S4; FunCat enrichments in Additional file 1). The observation emphasized the value of the integrative analysis of transcription and flux data that reflect the states of different functional levels of cells.

## Conclusions

In this study, the EMPath method for the detection of molecular interaction paths [13] was extended to allow for simultaneous utilization of transcriptome and fluxome data in an integrative manner. The method was applied to a combined network of *S. cerevisiae*’s metabolic and protein-protein interactions. In contrast to existing path finding methods [13,17-20,59], data from two sources were combined into one weighting scheme. Thus, the identification of potentially information transferring molecular paths beyond a single functional level of cells was enabled. The molecular paths coupled cellular components and processes distant at first sight but associated through different biochemical interactions with the oxygen-dependent phenotype changes in *S. cerevisiae*. New light was shed on the *S. cerevisiae* phenotypes previously investigated separately with transcription and on the level of *in vivo* fluxes [21-23]. However, it was observed that while the combined weighting scheme was of profound interest, all the three different weighting schemes resulted in enriched molecular paths providing complementary insight into the oxygen-dependent phenotypes of *S. cerevisiae*. In addition, certain processes were dominated by post-transcriptional level regulation i.e. glycolytic and fermentative fluxes were emphasized by the differences observed in the enriched molecular paths detected with the different weighting schemes. In particular, the detected molecular paths highlighted protein-protein interactions between the enzymes of central carbon metabolism that could possibly mediate coordinated post-transcriptional regulation of the differential *in vivo* activity of central metabolism in *S. cerevisiae* in two different respirofermentative metabolic states. Further, the down-regulation of oxidative stress in *S. cerevisiae* in conditions of 2.8% oxygenation compared to fully aerobic conditions was found to be related and potentially restricted to the down-regulation of peroxisomal activities. The results further suggested that a limited availability of oxygen and the consequently decreased respirative activity may affect transport reactions of *S. cerevisiae* across the mitochondrial membrane under conditions of 0.5% oxygen provision. Finally, the paths included metabolic interactions via metabolic intermediates in the crossroads of altered processes, such as acetyl-CoA and succinate, whose concentrations could be potential phenotypic markers.

## Abbreviations

ACHR: Artificial Centering Hit-and-Run; CER: Carbon Evolution Rate; COBRA: COnstraint-Based Reconstruction and Analysis; EMPath: Enriched Molecular Path detection; FDR: False Discovery Rate; FiDePa: Finding Deregulated Paths; FunCat: Functional protein Categories; GEO: Gene Expression Omnibus; GGEA: Gene Graph Enrichment Analysis; PPI: Protein-Protein Interaction; ROS: Reactive Oxygen Species; SEM: Standard Error of the Mean; SGD: *Saccharomyces cerevisiae* Genome Database; STRING: Search Tool for the Retrieval of Interacting Genes; TEAK: Topology Enrichment Analysis frameworK.

## Competing interest

The authors declare that they have no competing interest.

## Authors’ contributions

EL performed the path detection and enrichment analyses. PJ performed flux distribution sampling and interpreted the results. MeO designed the node weighting scheme. EL, PJ, and MeO conceived the study, and EL and PJ wrote the manuscript. ER conceived the biological questions for the study. MaO and MP supervised the study. All authors read and accepted the final manuscript.

## Supplementary Material

Additional file 1 The Description sheet contains detailed description of each supplementary table of this file. Click here for file

Additional file 2 **Detected molecular paths combined into one network.** Detected molecular paths combined into one network in the below mentioned figure numbers and comparisons. • **Figure S1.** Detected up-regulated paths combined into one network, 20.9% vs 2.8%, only flux data used^*^. • **Figure S2.** Detected down-regulated paths combined into one network, 20.9% vs 2.8%, only flux data used^*^. • **Figure S3.** Detected up-regulated paths combined into one network, 2.8% vs 0.5%, only flux data used^*^. • **Figure S4.** Detected down-regulated paths combined into one network, 2.8% vs 0.5%, only transcription data used^*^. * The most significant paths are aligned on the vertical axis. Squares are enzymatic reactions and triangles are signaling proteins. On the node labels transcriptomics weights are abbreviated by “tw” and fluxomics weights by “fw”. Also, the node labels contain gene names encoding a signaling protein/catalyzer and gene regulatory rules in which OR-operand is labeled by “|” and AND-operand by “&”. The following abbreviations are used in compartment names: “mit” = “mitochondrion”, “cyt” = “cytoplasm”, “ext” = “extracellular”, “er” = “endoplasmic reticulum”, “gol” = “golgi”, “per” = “peroxisome” and in metabolite names: “CoA” = “coenzyme A”, “cer” = “ceramide”. The nodes with positive total weight are colored by red, with negative total weight by blue and with neutral weight by grey. A few duplicate nodes (i.e. enzymes that have same encoding genes and catalyze separate reactions) are removed in order to prevent the visualization from getting too crowded. The red edges are protein-protein interactions in which STRING database [28] is abbreviated by “S” and kinase phosphatase interaction network [13] by “KPI”. The yellow edges are metabolic edges metabolic representing a shared substrate/product between two reactions. Click here for file

## References

[B1] Moxley JF Jewett MC Antoniewicz MR Villas-Boas SG Alper H Wheeler RT Tong L Hinnebusch AG Ideker T Nielsen J Stephanopoulos G Linking high-resolution metabolic flux phenotypes and transcriptional regulation in yeast modulated by the global regulator Gcn4p Proc Natl Acad Sci USA 2009 106 16 6477 6482 10.1073/pnas.0811091106 19346491 PMC2672541

[B2] Bordel S Agren R Nielsen J Sampling the solution space in genome-scale metabolic networks reveals transcriptional regulation in key enzymes PLoS Comput Biol 2010 6 7 1000859 10.1371/journal.pcbi.1000859 PMC2904763 20657658

[B3] Kim S Volsky D PAGE: parametric analysis of gene set enrichment BMC Bioinforma 2005 6 1 1 12 10.1186/1471-2105-6-1 PMC1183189 15941488

[B4] Patil KR Nielsen J Uncovering transcriptional regulation of metabolism by using metabolic network topology Proc Natl Acad Sci USA 2005 102 8 2685 2689 10.1073/pnas.0406811102 15710883 PMC549453

[B5] Barabási A Gulbahce N Loscalzo J Network medicine: a network-based approach to human disease Nat Rev Genet 2011 12 1 56 68 10.1038/nrg2918 21164525 PMC3140052

[B6] Chuang HY Lee E Liu YT Lee D Ideker T Network-based classification of breast cancer metastasis Mol Syst Biol 2007 3 1 140 17940530 10.1038/msb4100180 PMC2063581

[B7] Chuang H Rassenti L Salcedo M Licon K Kohlmann A Haferlach T Foà R Ideker T Kipps TJ Subnetwork-based analysis of chronic lymphocytic leukemia identifies pathways that associate with disease progression Blood 2012 120 13 2639 2649 10.1182/blood-2012-03-416461 22837534 PMC3460686

[B8] Nandy SK Jouhten P Nielsen J Reconstruction of the yeast protein-protein interaction network involved in nutrient sensing and global metabolic regulation BMC Syst Biol 2010 4 68 10.1186/1752-0509-4-68 20500839 PMC2889877

[B9] Breitkreutz A Choi H Sharom JR Boucher L Neduva V Larsen B Lin Z Breitkreutz B Stark C Liu G Ahn J Dewar-Darch D Reguly T Tang X Almeida R Qin ZS Pawson T Gingras A Nesvizhskii AI Tyers M A global protein kinase and phosphatase interaction network in yeast Science 2010 328 5981 1043 1046 10.1126/science.1176495 20489023 PMC3983991

[B10] Yu H Braun P Yıldırım MA Lemmens I Venkatesan K Sahalie J Hirozane-Kishikawa T Gebreab F Li N Simonis N Hao T Rual J Dricot A Vazquez A Murray RR Simon C Tardivo L Tam S Svrzikapa N Fan C De Smet A Motyl A Hudson ME Park J Xin X Cusick ME Moore T Boone C Snyder M Roth FP Barabási A Tavernier J Hill DE Vidal M High-quality binary protein interaction map of the yeast interactome network Science 2008 322 5898 104 110 10.1126/science.1158684 18719252 PMC2746753

[B11] Min Lee J Gianchandani EP Eddy JA Papin JA Dynamic analysis of integrated signaling, metabolic, and regulatory networks PLoS Comput Biol 2008 4-5 e1000086 18483615 10.1371/journal.pcbi.1000086 PMC2377155

[B12] Papin JA Palsson BO Topological analysis of mass-balanced signaling networks: a framework to obtain network properties including crosstalk J Theor Biol 2004 227 2 283 297 10.1016/j.jtbi.2003.11.016 14990392

[B13] Lindfors E Gopalacharyulu PV Halperin E Orešič M Detection of molecular paths associated with insulitis and type 1 diabetes in non-obese diabetic mouse PLoS ONE 2009 4-10 e7323 19798418 10.1371/journal.pone.0007323 PMC2749452

[B14] Alon N Yuster R Zwick U Color-coding J ACM 1995 42 844 856 10.1145/210332.210337

[B15] Scott J Ideker T Karp RM Sharan R Efficient algorithms for detecting signaling pathways in protein interaction networks J Comput Biol 2006 13 1 13 10.1089/cmb.2006.13.133 16597231

[B16] Dost B Shlomi T Gupta N Ruppin E Bafna V Sharan R QNet: a tool for querying protein interaction networks J Comput Biol 2008 15-7 913 925 18707533 10.1089/cmb.2007.0172

[B17] Geistlinger L Csaba G Küffner R Mulder N Zimmer R From sets to graphs: towards a realistic enrichment analysis of transcriptomic systems Bioinformatics 2011 27 i366 i373 10.1093/bioinformatics/btr228 21685094 PMC3117393

[B18] Keller A Backes C Gerasch A Kaufmann M Kohlbacher O Meese E Lenhof H A novel algorithm for detecting differentially regulated paths based on gene set enrichment analysis Bioinformatics 2009 25 21 2787 2794 10.1093/bioinformatics/btp510 19713416 PMC2781748

[B19] Judeh T Johnson C Kumar A Zhu D TEAK: topology enrichment analysis frameworK for detecting activated biological subpathways Nucleic Acids Res 2012 1 13 10.1093/nar/gks1299 PMC3561980 23268448

[B20] Martini P Sales G Massa MS Chiogna M Romualdi C Along signal paths: an empirical gene set approach exploiting pathway topology Nucleic Acids Res 2013 41 1 e19 e19 10.1093/nar/gks866 23002139 PMC3592432

[B21] Rintala E Toivari M Pitkanen J Wiebe M Ruohonen L Penttila M Low oxygen levels as a trigger for enhancement of respiratory metabolism in *Saccharomyces cerevisiae* BMC Genomics 2009 10 1 461 10.1186/1471-2164-10-461 19804647 PMC2767370

[B22] Wiebe MG Rintala E Tamminen A Simolin H Salusjärvi L Toivari M Kokkonen JT Kiuru J Ketola RA Jouhten P Huuskonen A Maaheimo H Ruohonen L Penttilä M Central carbon metabolism of *Saccharomyces cerevisiae* in anaerobic, oxygen-limited and fully aerobic steady-state conditions and following a shift to anaerobic conditions FEMS Yeast Res 2008 8 1 140 154 10.1111/j.1567-1364.2007.00234.x 17425669

[B23] Jouhten P Rintala E Huuskonen A Tamminen A Toivari M Wiebe M Ruohonen L Penttila M Maaheimo H Oxygen dependence of metabolic fluxes and energy generation of *Saccharomyces cerevisiae* CEN.PK113-1A BMC Syst Biol 2008 2 1 60 10.1186/1752-0509-2-60 18613954 PMC2507709

[B24] Jouhten P Wiebe M Penttilä M Dynamic flux balance analysis of the metabolism of Saccharomyces cerevisiae during the shift from fully respirative or respirofermentative metabolic states to anaerobiosis FEBS J 2012 279 18 3338 3354 10.1111/j.1742-4658.2012.08649.x 22672422

[B25] Herrgård M Swainston N Dobson P Dunn W Arga K Arvas M Blüthgen N Borger S Costenoble R Heinemann M Hucka M Le Novère N Li P Liebermeister W Mo M Oliveira A Petranovic D Pettifer S Simeonidis E Smallbone K Spasić I Weichart D Brent R Broomhead D Westerhoff H Kirdar B Penttilä M Klipp E Palsson B Sauer U Oliver S Mendes P Nielsen J Kell D A consensus yeast metabolic network reconstruction obtained from a community approach to systems biology Nat Biotechnol 2008 26 1155 1160 10.1038/nbt1492 18846089 PMC4018421

[B26] Dobson P Smallbone K Jameson D Simeonidis E Lanthaler K Pir P Lu C Swainston N Dunn W Fisher P Hull D Brown M Oshota O Stanford N Kell D King R Oliver S Stevens R Mendes P Further developments towards a genome-scale metabolic model of yeast BMC Syst Biol 2010 4 1 145 10.1186/1752-0509-4-145 21029416 PMC2988745

[B27] Szklarczyk D Franceschini A Kuhn M Simonovic M Roth A Minguez P Doerks T Stark M Muller J Bork P Jensen LJ Von Mering C The STRING database in 2011: functional interaction networks of proteins, globally integrated and scored Nucleic Acids Res 2011 39 Database-I 561 568 10.1093/nar/gkq973 PMC3013807 21045058

[B28] Barrett T Troup DB Wilhite SE Ledoux P Evangelista C Kim IF Tomashevsky M Marshall KA Phillippy KH Sherman PM Muertter RN Holko M Ayanbule O Yefanov A Soboleva A NCBI GEO: archive for functional genomics data sets—10 years on Nucleic Acids Res 2011 39 suppl 1 D1005 D1010 21097893 10.1093/nar/gkq1184 PMC3013736

[B29] Kaufman DE Smith RL Direction choice for accelerated convergence in hit-and-run sampling Oper Res January/February 1998 46 1 84 95 10.1287/opre.46.1.84

[B30] Becker SA Feist AM Mo ML Hannum G Palsson BO Herrgard MJ Quantitative prediction of cellular metabolism with constraint-based models: the COBRA Toolbox Nat Protocols 2007 2 3 727 738 10.1038/nprot.2007.99 17406635

[B31] Ruepp A Zollner A Maier D Albermann K Hani J Mokrejs M Tetko I Güldener U Mannhaupt G Münsterkötter M Mewes HW The FunCat, a functional annotation scheme for systematic classification of proteins from whole genomes Nucleic Acids Res 2004 32 18 5539 5545 10.1093/nar/gkh894 15486203 PMC524302

[B32] Benjamini Y Hochberg Y Controlling the false discovery rate: a practical and powerful approach to multiple testing J R Stat Soc 1995 57-1 289 300

[B33] Palmieri L Vozza A Hönlinger A Dietmeier K Palmisano A Zara V Palmieri F The mitochondrial dicarboxylate carrier is essential for the growth of Saccharomyces cerevisiae on ethanol or acetate as the sole carbon source Mol Microbiol 1999 31 2 569 577 10.1046/j.1365-2958.1999.01197.x 10027973

[B34] Dduntze W Neumann D Atzpodien W Holzer H Gancedo JM Studies on the regulation and localization of the glyoxylate cycle enzymes in Saccharomyces cerevisiae Eur J Biochem 1969 10 1 83 89 5345986 10.1111/j.1432-1033.1969.tb00658.x

[B35] Minard KI McAlister-Henn L Sources of NADPH in yeast vary with carbon source J Biol Chem 2005 280 48 39890 39896 10.1074/jbc.M509461200 16179340

[B36] Gebert N Gebert M Oeljeklaus S von der Malsburg K Stroud D Kulawiak B Wirth C Zahedi R Dolezal P Wiese S Simon O Schulze-Specking A Truscott K Sickmann A Rehling P Guiard B Hunte C Warscheid B van der Laan M Pfanner N Wiedemann N Dual function of Sdh3 in the respiratory chain and TIM22 protein translocase of the mitochondrial inner membrane Mol Cell 2011 44 5 811 818 10.1016/j.molcel.2011.09.025 22152483

[B37] Meinecke M Cizmowski C Schliebs W Kruger V Beck S Wagner R Erdmann R The peroxisomal importomer constitutes a large and highly dynamic pore Nat Cell Biol 2010 12 3 273 277 20154681 10.1038/ncb2027

[B38] Usaite R Jewett MC Oliveira AP Yates JR Olsson L Nielsen J Reconstruction of the yeast Snf1 kinase regulatory network reveals its role as a global energy regulator Mol Syst Biol 2009 5 1 319 19888214 10.1038/msb.2009.67 PMC2795470

[B39] Abdulrehman D Monteiro PT Teixeira MC Mira NP Lourenço AB Dos Santos SC Cabrito TR Francisco AP Madeira SC Aires RS Oliveira AL Sá-Correia I Freitas AT YEASTRACT: providing a programmatic access to curated transcriptional regulatory associations in Saccharomyces cerevisiae through a web services interface Nucleic Acids Res 2011 39 suppl 1 D136 D140 20972212 10.1093/nar/gkq964 PMC3013800

[B40] Monteiro PT Mendes ND Teixeira MC D’Orey S Tenreiro S Mira NP Pais H Francisco AP Carvalho AM Lourenço AB Sá-Correia I Oliveira AL Freitas AT YEASTRACT-DISCOVERER: new tools to improve the analysis of transcriptional regulatory associations in Saccharomyces cerevisiae Nucleic Acids Res 2008 36 suppl 1 D132 D136 18032429 10.1093/nar/gkm976 PMC2238916

[B41] Teixeira MC Monteiro P Jain P Tenreiro S Fernandes AR Mira NP Alenquer M Freitas AT Oliveira AL Sá-Correia I The YEASTRACT database: a tool for the analysis of transcription regulatory associations in Saccharomyces cerevisiae Nucleic Acids Res 2006 34 suppl 1 D446 D451 16381908 10.1093/nar/gkj013 PMC1347376

[B42] Koerkamp MG Rep M Bussemaker HJ Hardy GPMA Mul A Piekarska K Szigyarto CA De Mattos JMT Tabak HF Dissection of transient oxidative stress response in Saccharomyces cerevisiae by using DNA microarrays Mol Biol Cell 2002 13 8 2783 2794 10.1091/mbc.E02-02-0075 12181346 PMC117942

[B43] Paulsel AL Merz AJ Nickerson DP Vps9 family protein Muk1 is the second Rab5 guanosine nucleotide exchange factor in budding yeast J Biol Chem 2013 288 25 18162 18171 10.1074/jbc.M113.457069 23612966 PMC3689959

[B44] van den Berg MA De Jong-Gubbels P Kortland CJ Van Dijken JP Pronk JT Steensma HY The two Acetyl-coenzyme a synthetases of saccharomyces cerevisiae differ with respect to kinetic properties and transcriptional regulation J Biol Chem 1996 271 46 28953 28959 10.1074/jbc.271.46.28953 8910545

[B45] Chen Y Siewers V Nielsen J Profiling of cytosolic and peroxisomal acetyl-CoA metabolism in*Saccharomyces cerevisiae* PLoS ONE 2012 7 8 e42475 10.1371/journal.pone.0042475 22876324 PMC3411639

[B46] Sickmann A Reinders J Wagner Y Joppich C Zahedi R Meyer HE Schönfisch B Perschil I Chacinska A Guiard B Rehling P Pfanner N Meisinger C The proteome of *Saccharomyces cerevisiae* mitochondria Proc Natl Acad Sci USA 2003 100 13207 13212 10.1073/pnas.2135385100 14576278 PMC263752

[B47] Epstein C Waddle J Hale W4 Davé V Thornton J Macatee T Garner H Butow R Genome-wide responses to mitochondrial dysfunction Mol Biol Cell 2001 12 2 297 308 10.1091/mbc.12.2.297 11179416 PMC30944

[B48] Saint-Prix F Bönquist L Dequin S Functional analysis of the ALD gene family of Saccharomyces cerevisiae during anaerobic growth on glucose: the NADP + -dependent Ald6p and Ald5p isoforms play a major role in acetate formation Microbiology 2004 150 7 2209 2220 10.1099/mic.0.26999-0 15256563

[B49] Grabowska D Chelstowska A The ALD6 gene product is indispensable for providing NADPH in yeast cells lacking glucose-6-phosphate dehydrogenase activity J Biol Chem 2003 278 16 13984 13988 10.1074/jbc.M210076200 12584194

[B50] Grilley MM Stock SD Dickson RC Lester RL Takemoto JY Syringomycin action gene SYR2 is essential for sphingolipid 4-hydroxylation in saccharomyces cerevisiae J Biol Chem 1998 273 18 11062 11068 10.1074/jbc.273.18.11062 9556590

[B51] Dunn TM Haak D Monaghan E Beeler TJ Synthesis of monohydroxylated inositolphosphorylceramide (IPC-C) in Saccharomyces cerevisiae requires Scs7p, a protein with both a cytochrome b5-like domain and a hydroxylase/desaturase domain Yeast 1998 14 4 311 321 10.1002/(SICI)1097-0061(19980315)14:4<311::AID-YEA220>3.0.CO;2-B 9559540

[B52] Dickson RC Roles for sphingolipids in Saccharomyces cerevisiae Adv Exp Med Biol 2010 688 217 231 10.1007/978-1-4419-6741-1_15 20919657 PMC5612324

[B53] Luttik MAH Kötter P Salomons FA van der Klei IJ Van Dijken JP Pronk JT The saccharomyces cerevisiae ICL2 gene encodes a mitochondrial 2-methylisocitrate lyase involved in propionyl-coenzyme a metabolism J Bacteriol 2000 182 24 7007 7013 10.1128/JB.182.24.7007-7013.2000 11092862 PMC94827

[B54] Collins SR Kemmeren P Zhao X Greenblatt JF Spencer F Holstege FCP Weissman JS Krogan NJ Toward a comprehensive atlas of the physical interactome of saccharomyces cerevisiae Mol Cell Proteomics 2007 6 3 439 450 17200106 10.1074/mcp.M600381-MCP200

[B55] Cankorur-Cetinkaya A Dereli E Eraslan S Karabekmez E Dikicioglu D Kirdar B A novel strategy for selection and validation of reference genes in dynamic multidimensional experimental design in yeast PLoS ONE 2012 6 e38351 22675547 10.1371/journal.pone.0038351 PMC3366934

[B56] Bruckmann A Hensbergen PJ Balog CI Deelder AM de Steensma HY Van Heusden GP Post-transcriptional control of the saccharomyces cerevisiae proteome by 14–3–3 proteins J Proteome Res 2007 5 1689 17397208 10.1021/pr0605522

[B57] Bodenmiller B Campbell D Gerrits B Lam H Jovanovic M Picotti P Schlapbach R Aebersold R PhosphoPep[mdash]a database of protein phosphorylation sites in model organisms Nat Biotech 2008 26 12 1339 1340 10.1038/nbt1208-1339 PMC2743685 19060867

[B58] Oliveira AP Ludwig C Picotti P Kogadeeva M Aebersold R Sauer U Regulation of yeast central metabolism by enzyme phosphorylation Mol Syst Biol 2012 8 1 623 23149688 10.1038/msb.2012.55 PMC3531909

[B59] Kim Y Wuchty S Przytycka TM Identifying causal genes and dysregulated pathways in complex diseases PLoS Comput Biol 2011 7 3 e1001095 10.1371/journal.pcbi.1001095 21390271 PMC3048384

