# Supplementary material for: Integration of transcription and flux data reveals molecular paths associated with differences in oxygen-dependent phenotypes of Saccharomyces cerevisiae
Source: BMC Syst Biol. 2014 Feb 14;8:16. doi: 10.1186/1752-0509-8-16 (PMC3930817; doi:10.1186/1752-0509-8-16)
Supplement: Additional file 2 — Detected molecular paths combined into one network. Detected molecular paths combined into one network in the below mentioned figure numbers and comparisons. • Figure S1. Detected up-regulated paths combined into one network, 20.9% vs 2.8%, only flux data used*. • Figure S2. Detected down-regulated paths combined into one network, 20.9% vs 2.8%, only flux data used*. • Figure S3. Detected up-regulated paths combined into one network, 2.8% vs 0.5%, only flux data used*. • Figure S4. Detected down-regulated paths combined into one network, 2.8% vs 0.5%, only transcription data used*. * The most significant paths are aligned on the vertical axis. Squares are enzymatic reactions and triangles are signaling proteins. On the node labels transcriptomics weights are abbreviated by “tw” and fluxomics weights by “fw”. Also, the node labels contain gene names encoding a signaling protein/catalyzer and gene regulatory rules in which OR-operand is labeled by “|” and AND-operand by “&”. The following abbreviations are used in compartment names: “mit” = “mitochondrion”, “cyt” = “cytoplasm”, “ext” = “extracellular”, “er” = “endoplasmic reticulum”, “gol” = “golgi”, “per” = “peroxisome” and in metabolite names: “CoA” = “coenzyme A”, “cer” = “ceramide”. The nodes with positive total weight are colored by red, with negative total weight by blue and with neutral weight by grey. A few duplicate nodes (i.e. enzymes that have same encoding genes and catalyze separate reactions) are removed in order to prevent the visualization from getting too crowded. The red edges are protein-protein interactions in which STRING database [28] is abbreviated by “S” and kinase phosphatase interaction network [13] by “KPI”. The yellow edges are metabolic edges metabolic representing a shared substrate/product between two reactions. [file 1752-0509-8-16-S2.pdf]

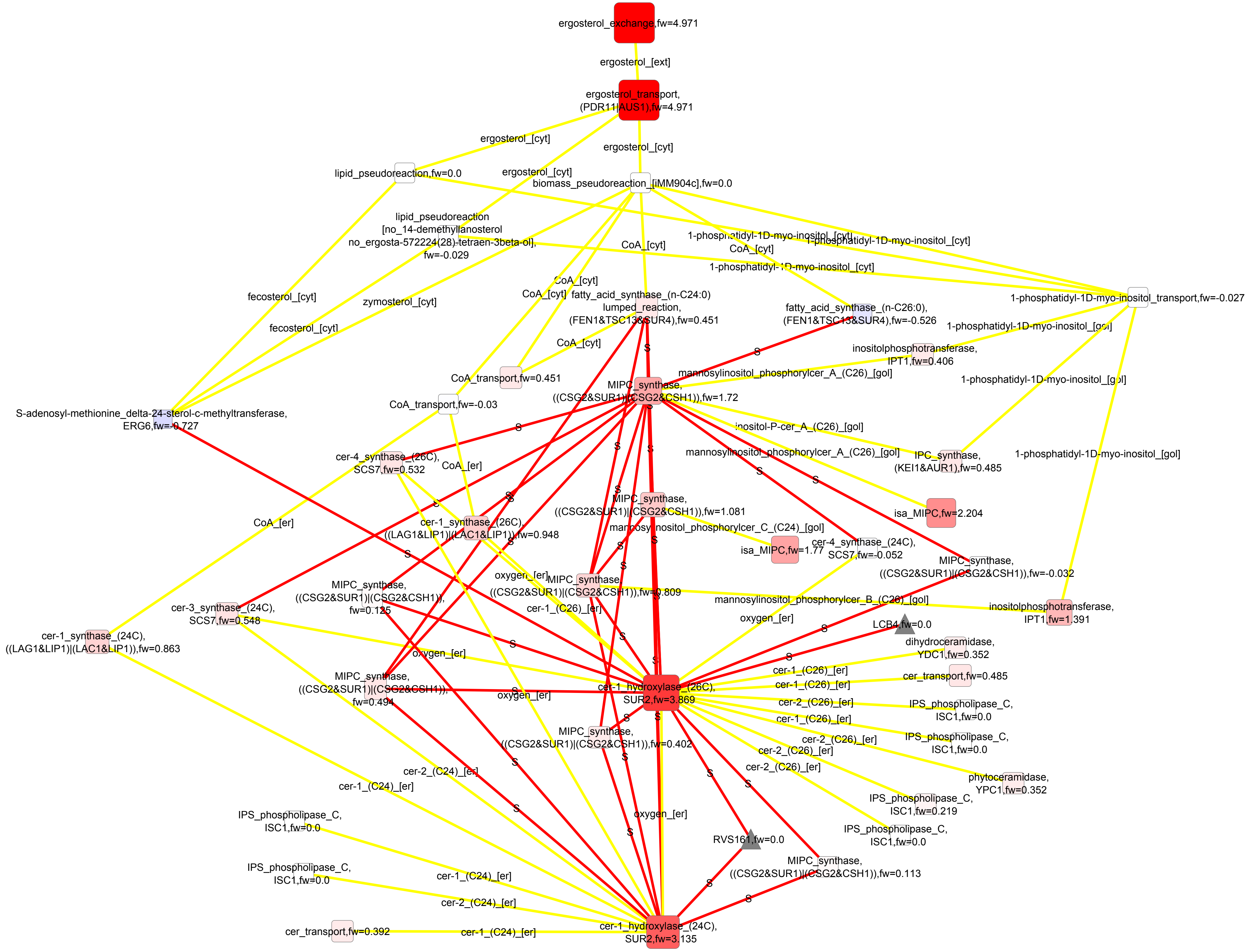

Figure S1 - Detected up-regulated molecular paths combined into one network, 20.9%vs2.8%, only fluxomics data used.

These paths are visualized by using the rules described on the legend of Figure 2.

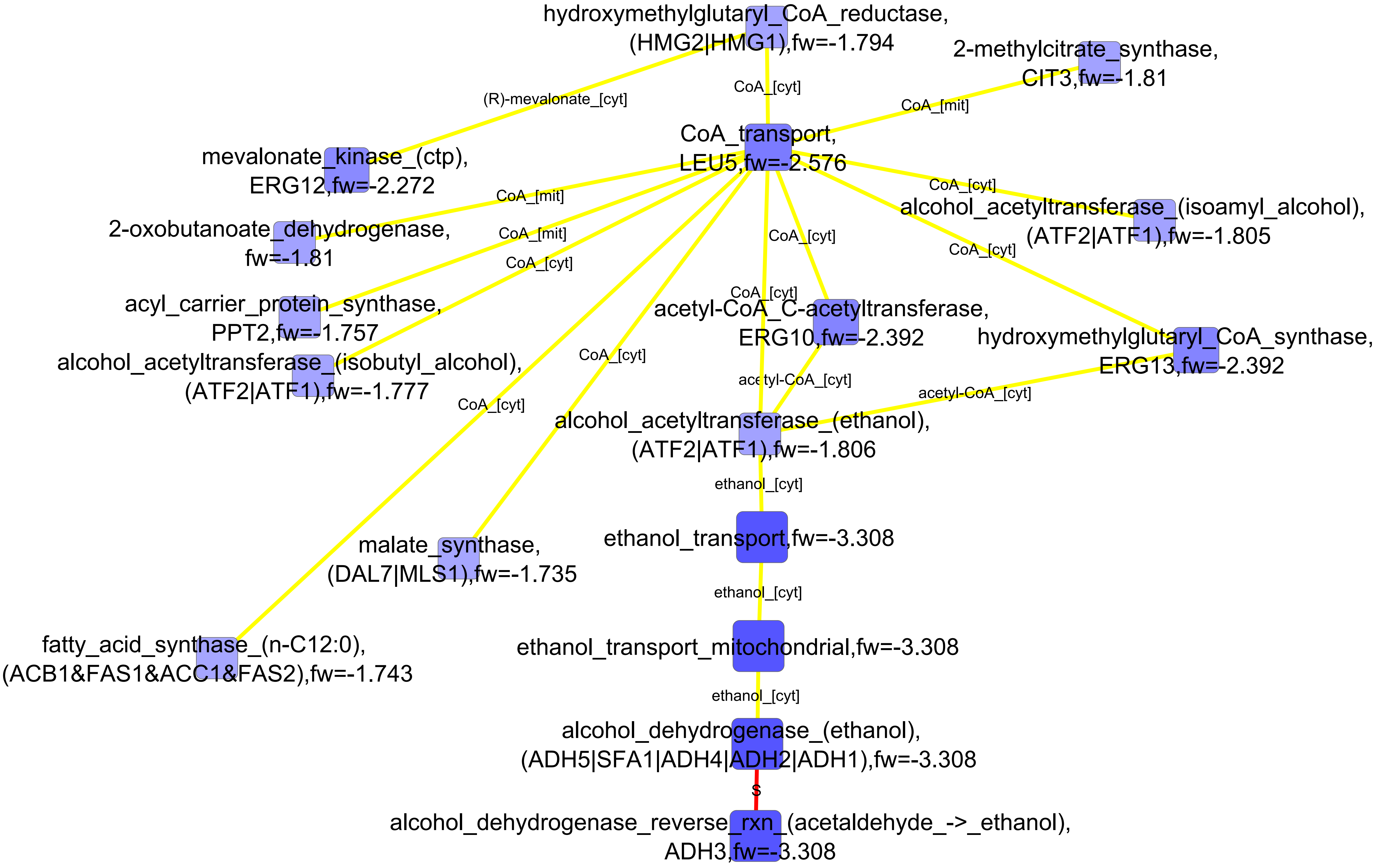

Figure S2 - Detected down-regulated molecular paths combined into one network, 20.9%vs2.8%, only fluxomics data used.

These paths are visualized by using the rules described on the legend of Figure 2.

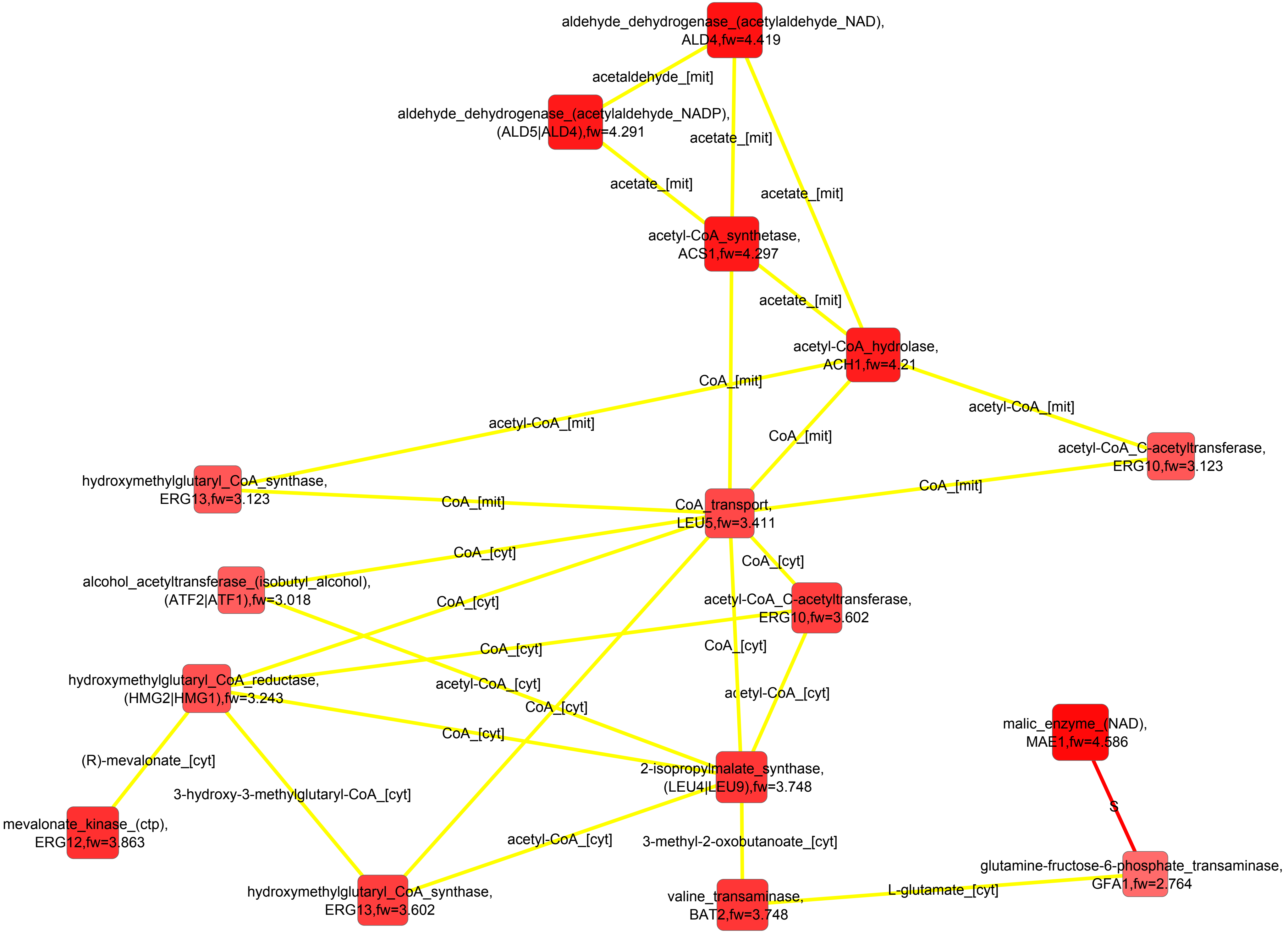

Figure S3 - Detected up-regulated molecular paths combined into one network, 2.8%vs0.5%, only fluxomics data used.

These paths are visualized by using the rules described on the legend of Figure 2.

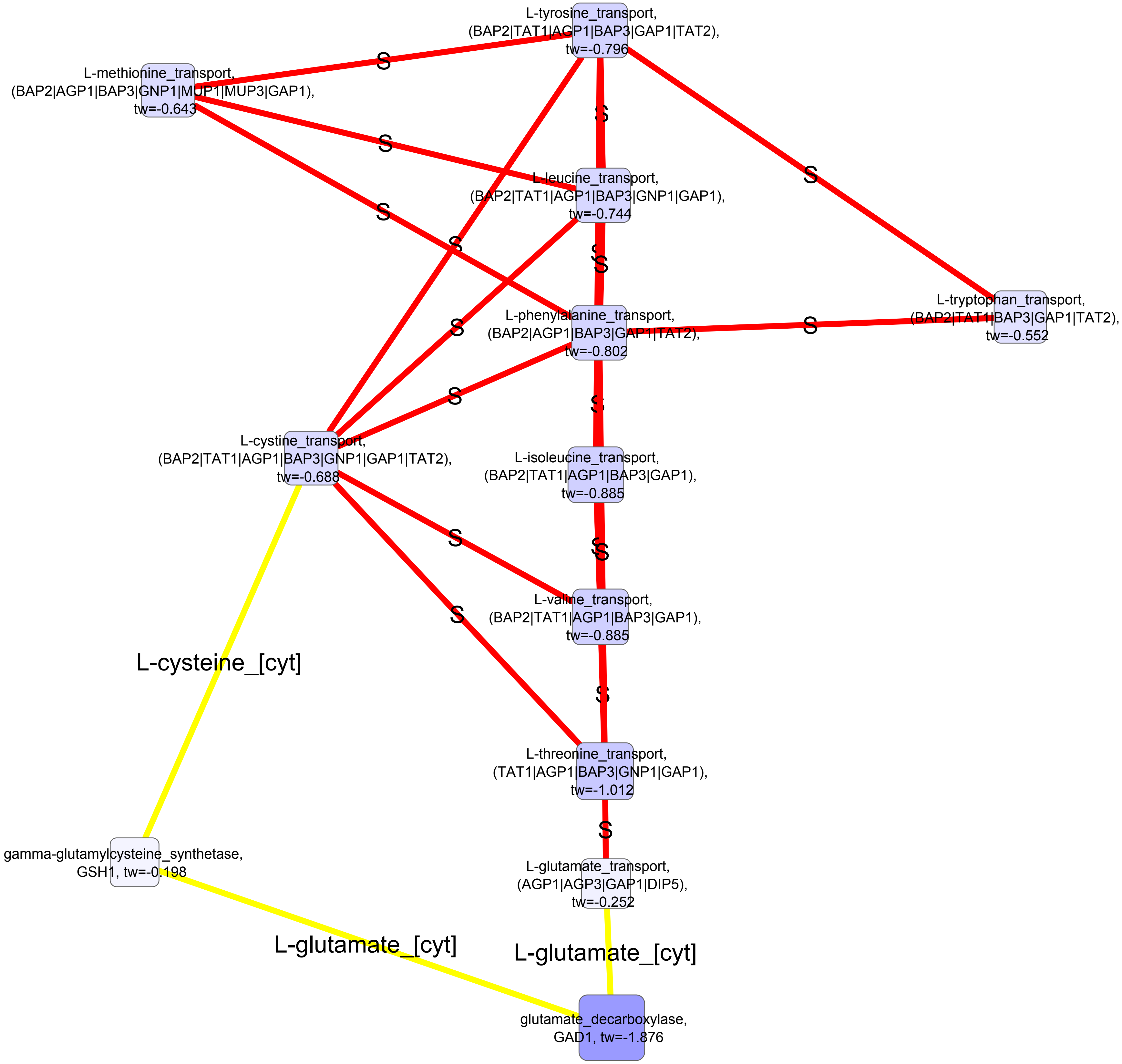

Figure S4 - Detected down-regulated molecular paths combined into one network, 2.8%vs0.5%, only transcriptomics data used.

These paths are visualized by using the rules described on the legend of Figure 2.
